# Fiber Optic-Based Thermal Integrity Profiling of Drilled Shaft: Inverse Modeling for Spiral Fiber Deployment Strategy

**DOI:** 10.3390/ma14185377

**Published:** 2021-09-17

**Authors:** Wen Deng, Ruoyu Zhong, Haiying Ma

**Affiliations:** 1 School of Civil Engineering, Southeast University, Nanjing 210096, China; wendeng@seu.edu.cn; 2 Department of Civil, Architectural and Environmental Engineering, Missouri University of Science and Technology, Rolla, MO 65409, USA; rzymh@mst.edu; 3 Department of Bridge Engineering, Tongji University, Shanghai 200082, China

**Keywords:** thermal integrity profiling, drilled shaft, fiber optic sensors, fiber deployment, inverse modeling

## Abstract

The current state of practice to interpret the thermal integrity profiling (TIP) data of drilled shaft is the so-called effective radius method. It uses the concrete pouring log and average temperature to construct a relationship between temperature distribution and effective radius that can be used to reconstruct a drilled shaft model. While this effective radius method is computationally inexpensive and has good operationality, it is not good at predicting the dimensions and shape of shaft defects. Upgrading the sensor used in conventional TIP from thermocouples/thermal wires to fiber optic sensors, the spatial resolution of the measured temperature will be enhanced. By using the newly proposed spiral fiber deployment strategy, we can improve the reconstruction of shaft defects in the integrity testing of drilled shafts. The corresponding inverse modeling of defected shaft reconstruction for spiral deployment is proposed in this paper based on the temperature distribution pattern that is learned from forward modeling. Through inverse modeling, the details of defects in drilled shafts can be reconstructed numerically. An analysis of the results shows that the prediction by inverse modeling has good agreement with the forward modeling set up initially. This work helps the evolution of the TIP from the nondestructive testing stage to the quantitative nondestructive evaluation stage.

## 1. Introduction

Drilled shafts are frequently used in the deep foundation construction of the transportation infrastructure of the world. The integrity of drilled shafts has a direct impact on the safety, durability and long-term performance of the overall infrastructure system. Thermal integrity profiling (TIP) testing [1] is an emerging testing methodology that measures temperatures with respect to depth within the drilled shaft. It was introduced recently within the past few years and has progressed from demonstration projects to more practical use [2,3,4,5]. This TIP method also shows good agreement with other nondestructive integrity testing methods [6]. The essential mechanism of TIP is to make use of the hydration heat to test the structural integrity. Defects would be shown as temperature anomalies in the measured temperature distribution field of poured concrete. This method enlarges the testing area from the concrete enclosed by the reinforcement cage to the concrete outside the reinforcement cage. Many states’ Departments of Transportation (DOTs) in the United States, such as FDOT, IDOT, Iowa DOT, WisDOT, and MoDOT, either were or are conducting and soliciting TIP research studies. The current temperature measurement of TIP mainly relies on either infrared probes or thermal wires/couples [7]. For example, the spatial resolution used for thermal wires was 30.48 cm (i.e., 1 ft) [7]. Based on these temperature measurement technologies, the spatial resolution of temperature measurement is limited. Due to this limitation, the current state of practice in TIP is to use concrete pouring log and average temperature measured to conduct a relation between average temperature and average radius [1,8] which is referred to as the effective radius method in this paper. When using this method to reconstruct the defected shaft, the reconstructed shaft is in a varying radius cylindrical shape. The actual location and shape of defects cannot be reconstructed by this equivalent radius method. Therefore, the impact of the defected shaft on the infrastructure cannot be estimated accurately or efficiently.

**Figure 1:**
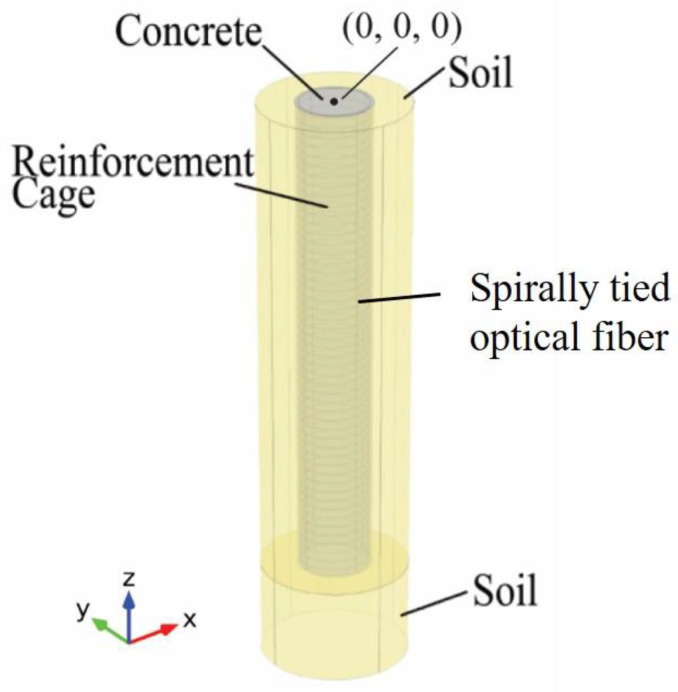
Shaft-soil model with spirally tied optical fiber on the reinforcement cage and surrounded by soil. Figure modified from [23].

With the development of sensing technology, the operationality, resolution, and cost of these temperature measurement methods can still be improved [8,9]. Distributed fiber optic sensors (DFOS) are currently regarded as a cutting-edge technology in many fields of civil engineering infrastructure sensing, such as bridge monitoring [10,11,12,13], slope and landslides [14,15,16,17], soil retaining walls [18,19], and tunnel monitoring [20,21]. Following on from these successful applications of DFOS in civil engineering infrastructure, fiber optic-based temperature measurement is proposed to improve the temperature measurement method of the conventional TIP. According to the current technology of fiber optic by using optical frequency domain reflectometry (OFDR), every point on a single optical fiber serves as a temperature sensor, and the spatial resolution of temperature measurements can be as high as every 0.5 mm in the practical applications with about 1 °C (i.e., 1.8 °F) temperature accuracy [22]. Moreover, the cost of materials of optical fiber can be minimal compared to infrared probes or thermocouples. By tying the optical fiber with metal sleeves spirally on the reinforcing cage [23], very high-resolution temperature distribution data through optical-fiber-based TIP can be obtained (Figure 1). Therefore, it can better predict the dimensions and location of defects through further numerical simulation. To keep our focus on the problem, a detailed introduction of the theory and configuration of OFDR is not given in this paper.

Fiber optic sensors have previously been introduced for TIP with a spatial resolution of 5 cm using Brillouin optical time domain reflectometry (BOTDR) technology [8,9], and the way to install fiber optic sensors by tying optical fibers in straight lines was still similar to using thermal wires for TIP. It prevents this fiber optic sensing from obtaining more comprehensive temperature data. Therefore, the reconstruction of drilled shafts cannot go beyond the effective radius method. Shaft defects such as voids cannot be reconstructed through this straight-line fiber deployment [8,9] and the estimation of the shaft integrity is thus compromised. By tying the optical fiber with a metal sleeve spirally on the reinforcing cage, high-spatial-resolution temperature distribution data through fiber optic-based TIP can be obtained (Figure 1) [23]. The small interval of the fiber optic sensor and the deployment approach within shafts can give a more comprehensive temperature distribution since fiber optic-based TIP obtains temperature data on a three-dimensional (3D) cylindrical surface instead of the two-dimensional straight lines we get from conventional TIP using infrared sensor or thermal wires. Although this new type of fiber deployment within the shaft can cause more challenges to maintain the integrity of the optical fibers and lead to other defects due to aggregate flow problems during shaft production, the rewarding enhancement of TIP capability might pay these challenges and difficulties off. Whether other distributed temperature sensing is suitable for this application of spiral deployment depends on the spatial resolution. The high spatial resolution is required for our proposed strategy to tie the optical fibers spirally around the reinforcement cage in the practical applications.

Based on this improvement in the temperature measurement of fiber optic-based TIP and newly proposed spiral deployment strategy, this paper aims to propose a new finite-element-method (FEM)-based inverse modeling for numerical reconstruction of the location and shape of defects within the drilled shaft. This more comprehensive reconstruction of the drilled shaft cannot be achieved through conventional TIP due to a lack of sufficient 3D distributed temperature data. This lack of field data will be tackled in our future work. To develop this inverse modeling method, the forward modeling is firstly used to obtain the temperature data spirally on the reinforcement cage surface as the virtual data of fiber optic sensing. These temperature data are used to develop inverse modeling through a trial-and-error method by placing the guessed defect in the model to match the forward modeling data. A rule is developed to facilitate the trial-and-error process to reconstruct the shape, location and size of the defect within the shaft. This inverse modeling will be tested and improved once the field data are available in our future work. The success of the research will greatly advance the construction technology used by state DOTs by enhancing the safety of the transportation system at a reduced cost of construction.

## 2. Methodology

### 2.1. Governing Equations

The essential mechanism of TIP relies on the heat generated by hydration process of concrete. In the scenario of drilled shaft integrity testing, the predominant physics involved is heat conduction as the major heat transport approach. Thus, we use heat conduction in a solid module of a commercial FEM-based software, COMSOL Multiphysics^®^, to run the simulation to acquire the temperature (*T*) field in the concrete shaft. The governing equation of heat conduction can be given by:

(1) $$\frac{\rho C_{p}\partial T}{\partial t}=\left[\frac{\partial }{\partial x}\left(k\frac{\partial T}{\partial x}\right)+\frac{\partial }{\partial y}\left(k\frac{\partial T}{\partial y}\right)+\frac{\partial }{\partial z}\left(k\frac{\partial T}{\partial z}\right)\right]+Q$$

where *C_p_* represents the heat capacity of the material; *k* is the thermal conductivity of the material; and *Q* is the heat source, which is mainly due to the hydration process within the concrete.

### 2.2. Heat Production

The heat production by the hydration process is the key factor of TIP. The combination of heat production and dissipation when defects are present could result in a temperature anomaly in the measured temperature distribution. Thus, an accurate description of heat production is critical in the numerical reconstruction of the drilled concrete shaft. The total amount and the rate of heat produced together determine the temperature distribution within the drilled shaft at a specific time. The amount and the rate of heat produced are related to the ingredients of the concrete. We employ equations given by Schindler and Folliard [24] to calculate the total heat production:

(2) $$Q=Q_{cem}p_{cem}+461p_{slag}+Q_{FA}p_{FA}$$

(3) $$Q_{cem}=500p_{C_{3}S}+260p_{C_{2}S}+866p_{C_{3}A}+420p_{C_{4}AF}+624p_{SO_{3}}+1186p_{FreeCaO}+850p_{MgO}$$

(4) $$Q_{FA}=1800p_{FACaO}$$

where *p* with different subscripts represents the weight ratio of each compound in terms of the total cementitious content and *Q* with different subscripts represents the heat generated according to each compound of the concrete. The subscripts represent the name of each compound: *cem* denotes the cement compound; *slag* denotes the slag compound; *FA* denotes the fly ash compound; *C*_3_*S* denotes the tricalcium silicate compound; *C*_2_*S* denotes the dicalcium silicate compound; *C*_3_*A* denotes the tricalcium aluminate compound; *C*_4_*AF* denotes the tetracalcium aluminoferrite compound; *SO*_3_ denotes the *SO*_3_ compound; *FreeCaO* denotes the free calcium oxide compound; and *MgO* denotes the magnesium oxide. *p_FACaO_* denotes the weight ratio of the calcium oxide content of the fly ash. The chemical composition of cement and fly ash are usually available from the supplier.

The degree of hydration can be determined by the following equation [1,24]:

(5) $$\alpha \left(t_{e}\right)=\alpha_{u}\exp\left(-{\left[\frac{\tau }{t_{e}}\right]}^{\beta }\right)$$

(6) $$\alpha_{u}=\frac{\left(1.031w/cm\right)}{\left(0.194+w/cm\right)}+0.5p_{FA}+0.3p_{SLAG}<1$$

(7) $$\beta =p_{C_{3}S}^{0.227}\cdot 181.4\cdot p_{C_{3}A}^{0.146}\cdot Blaine^{-0.535}\cdot p_{SO_{3}}^{0.558}\cdot \exp\left(-0.647p_{SLAG}\right)$$

(8) $$\tau =p_{C_{3}S}^{-0.401}\cdot 66.78\cdot p_{C_{3}A}^{-0.154}\cdot Blaine^{-0.804}\cdot p_{SO_{3}}^{-0.758}\cdot \exp\left(2.187\cdot p_{SLAG}+9.5\cdot p_{FA}\cdot p_{FACaO}\right)$$

where α(t) denotes the degree of hydration of cement at equivalent age *t_e_*; and w/cm is a water-cement ratio; *β* and *τ* are determined by the cementitious constituent fractions; *Blaine* denotes Blaine value, specific surface arear of cement (m^2^/kg). According to ASTM D7949-14, the recommended timing to perform TIP would be 12 h after concrete pouring until the number of days equivalent to the foundation diameter in meters divided by 0.3.

### 2.3. Heat Conductivity

In the drilled shaft model, heat dissipates into the air on the top of the shaft and at the surrounding soil in the field test. To take this into account, the heat flux boundary condition is defined at the top of the shaft and at the surrounding soil, and the temperature is set to room temperature, 23 °C (i.e., 73.4 °F), at the boundary for the demonstration of this inverse method. In this situation, since no gas or liquid is involved in the model, heat conduction is the major heat transport mechanism in the solid. Heat conductivity and heat capacity work together for this heat conduction process.

Soil as a multi-phase material consists of solids, air, and water. The specific value of thermal conductivity k of soil is determined by the constitution of soil and the thermal conductivity of each phase. To simplify the model, we consider soil a one-phase material and use equivalent thermal conductivity as its properties. The equivalent thermal conductivity can be determined by [24,25,26]:

(9) $$k_{1}=k_{s}-n\left[k_{s}-S_{w}k_{w}-\left(1-S_{w}\right)k_{a}\right]$$

where *k* with subscripts represents the thermal conductivity of each phase of soil; *n* is the porosity; and *S_w_* is the degree of water saturation. The subscript *s* denotes solid phase; *w* denotes water phase; and *a* denotes air phase.

A shape factor $\chi =\sqrt{S_{w}}$ is introduced into the equation to represent the effect caused by the shape of the void. Then, the equation becomes:

(10) $$k=\sqrt{S_{w}}\left\{k_{s}-n\left[k_{s}-S_{w}k_{w}-\left(1-S_{w}\right)k_{a}\right]\right\}+\left(1-\sqrt{S_{w}}\right)k_{a}$$

### 2.4. Heat Capacity

We assume the temperature of the soil is the same among three phases, and the heat capacity of the soil is also related to the three phases of the soil. The heat required to raise the temperature of the soil one kelvin can be calculated as the sum of the heat to raise one-degree of three phases separately, which in equation would be *C_s_m_s_* + *C_w_m_w_* + *C_g_m_g_*. The total weight of the soil is *m_s_* + *m_w_* + *m_g_*. Therefore, the value of soil heat capacity can the determined as follows [27]:

(11) $$C_{p}=\frac{C_{s}m_{s}+C_{w}m_{w}+C_{g}m_{g}}{m_{s}+m_{w}+m_{g}}$$

where *C* with subscripts represents the heat capacity of each phase of soil and *m* with subscripts represents the mass of each phase of soil.

Considering that the mass of air is negligible, the equation can be simplified as:

(12) $$C_{p}=\frac{C_{s}+C_{w}w}{1+w}$$

where *w* is water content.

### 2.5. Simulation Parameters

The model consists of three parts: the drilled concrete shaft, the soil surrounding the shaft, and the soil below the shaft. The diameter of the shaft is set to 1.83 m (i.e., 6 ft), and the entire length of the shaft is set to 15 m. In this model, to keep problem-focused, the reinforcement cage is assumed to align perfectly, although the alignment issue can also be discovered and adjusted through TIP analysis [28]. Due to the low heat capacity, high thermal conductivity, and the relatively small volume of the reinforcement cage, the reinforcement cage as well as the optical fibers with a metal sleeve are not considered modeling elements in our simulation. Even though there is no actual reinforcement cage being input to the model, the location of the reinforcement cage is still prescribed as a reference for optical fiber deployment. To measure the temperature distribution within a concrete shaft, optical fibers must be deployed inside the concrete shaft. To further improve the spatial resolution of TIP data, the optical fiber is chosen to be deployed spirally around the reinforcement cage so that the temperature along the optical fiber can be obtained. The vertical interval of virtual optical fiber tied spirally is 300 mm with an approximate tangent slope of 0.052. The thickness of soil outside the concrete shaft is as large as the diameter of the concrete shaft, which is 1.83 m. The numerical sampling resolution along the virtual optical fiber is set to 2 cm. The properties of concrete and soil surrounding concrete are listed in Table 1.

**Figure 2:**
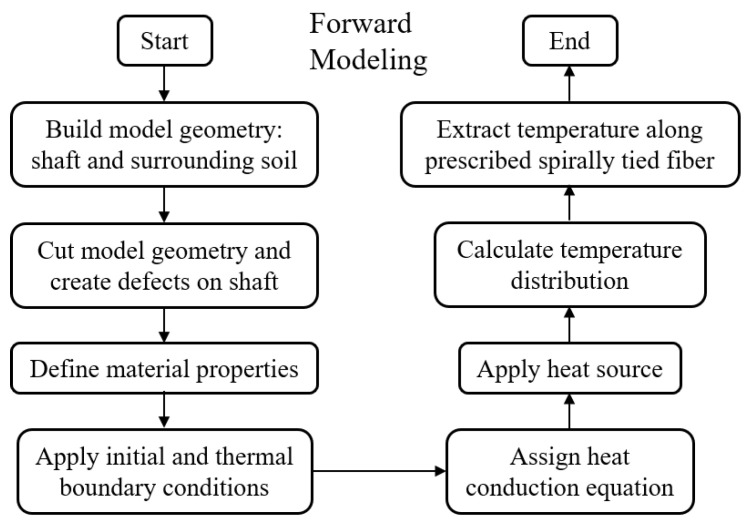
Simulation flowchart of the forward modeling for fiber optic-based TIP with spiral deployment.

**Figure 3:**
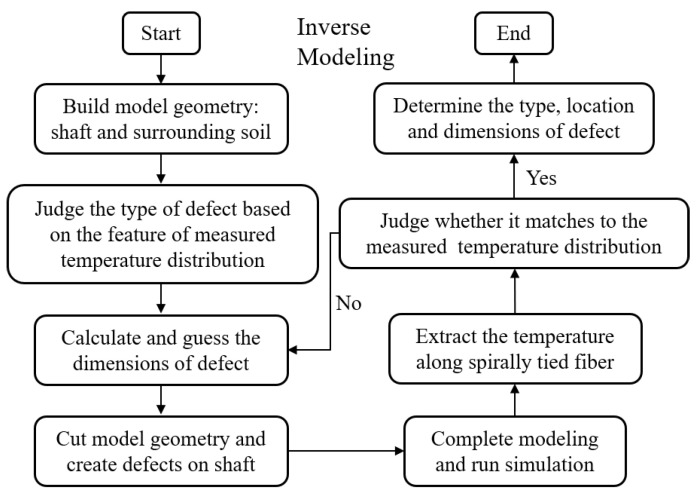
Simulation flowchart of the inverse modeling for fiber optic-based TIP with spiral deployment.

This simulation is conducted using FEM based commercial software COMSOL Multiphysics^®^, and the procedure of simulation can be referred to the flowcharts: Figure 2 is for forward modeling; Figure 3 is for inverse modeling. The mesh type is free tetrahedral mesh by setting the minimum element size to 20 mm. Several defects are set on the shaft for the forward modeling to generate temperature data for the subsequent inverse modeling.

## 3. Results and Discussion

In this section, results from the simulations of different defective concrete shafts are presented. The defects include two types: necking and voids [31]. Firstly, the forward modeling is considered. What follows is the proposed inverse modeling method along with examples of defect prediction using this inverse modeling. Bulge is not discussed in this study since it is similar to necking but with the opposite temperature anomaly. The method developed for necking also works for the bulge case, as well.

### 3.1. Necking Defect

#### 3.1.1. Forward Modeling

**Figure 4:**
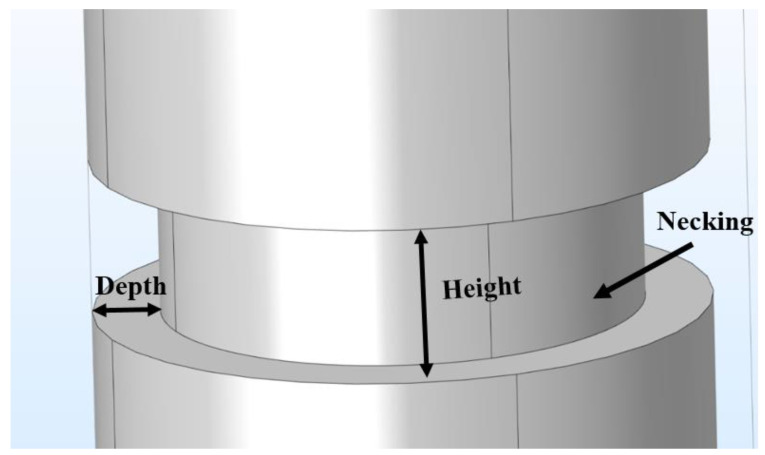
Sketch of necking defect for forward and inverse modeling.

**Figure 5:**
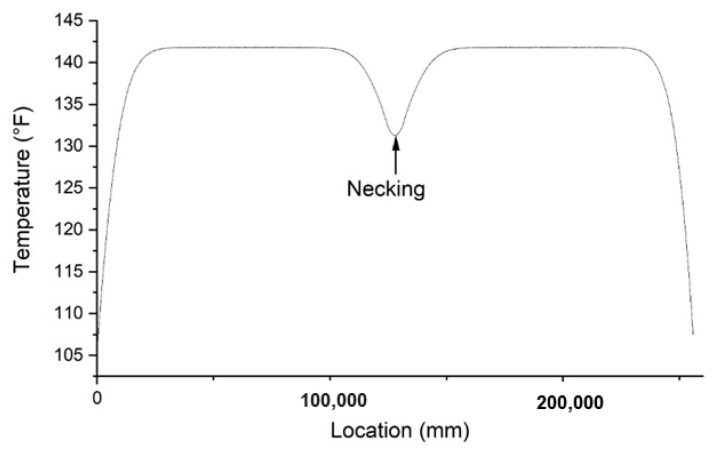
Temperature distribution on the optical fiber with necking defect. The location on the x-axis is given as the length of the virtual optical fiber tied spirally around the virtual reinforcement cage within the shaft.

The necking of the drilled shaft (Figure 4) is defined as a rapid reduction in the cross section of the shaft. In the simulation, necking is presented as a cylindrical section with a smaller radius connected to two cylindrical sections of the intact shaft. The existence of necking means there will be less hydration heat produced during the hydration process at that location. This necking defect can result in a region where the temperature is lower than upward and downward vicinity regions. The prescribed necking location for the forward modeling is set from 7.3 m to 7.7 m distant from the top of the shaft, and therefore the height of necking (Figure 4) is 400 mm. The depth of necking (Figure 4) is set to 200 mm. The temperature distribution along the prescribed optical fiber of the forward modeling of necking is presented in Figure 5. As shown in Figure 5, the temperature at the beginning (i.e., corresponding to the top of the shaft) and end (i.e., corresponding to the bottom of the shaft) of the optical fiber is smaller than that in the middle. The reason is that heat can dissipate from the top to the air and from the bottom to the soil, and the temperature is thus higher in the middle of shaft. At the middle of the plot, there is a region that shows a low-temperature anomaly. That is the location of necking in the defective concrete shaft.

Only one local minimum on the plot can be seen in Figure 5, which means that there is only a necking defect in the shaft. To separate necking from the void defect, one can calculate the length of the optical fiber that passes a single temperature drop in the plot. If the length is larger than one or two perimeters of the reinforcement cage with only one temperature drop, the defect can be interpreted as necking. If several temperature drops are presented continuously on the plot, one can interpret it as a void defect.

#### 3.1.2. Inverse Modeling

The location of necking can be estimated using the location of the local temperature minimum shown in the plot. Optical fibers tied spirally around the reinforcement cage should pass through the affected region by necking. As it passes through the necking region, the temperature measurement reaches the local minimum in the plot. The depth and azimuthal angle of necking can be calculated based on the *x* coordinate of the local temperature minimum on the plot as shown in Figure 5. By knowing the deployment detail of the optical fiber, the *x* coordinate representing the length of the optical fiber can be converted to the exact location within the concrete shaft, and therefore the location of the defect can be deduced.

**Figure 6:**
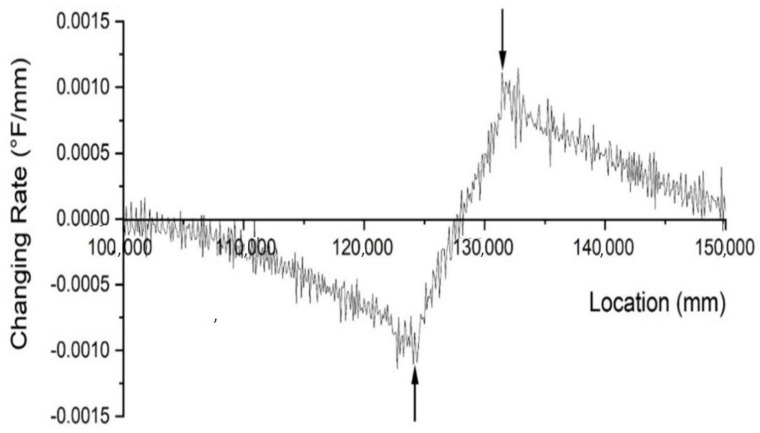
Temperature change rate along the optical fiber for the necking defect.

**Figure 7:**
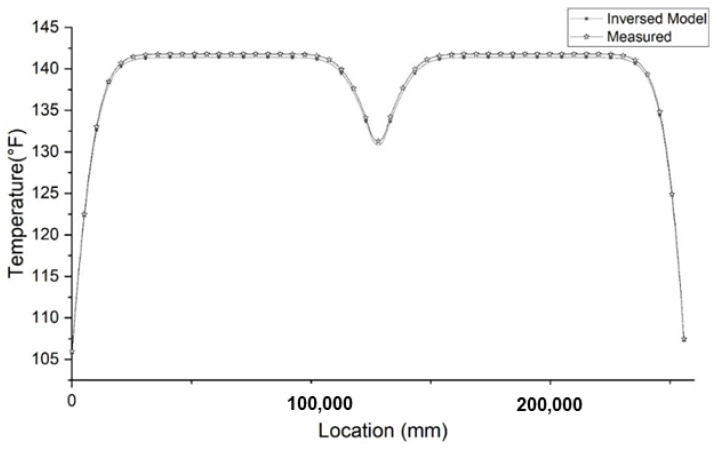
Comparison of measured temperature and inverse modeling for the necking defect.

To reconstruct the defect, aside from the location, the size of the defect is also a critical factor. The size of the necking is determined by two parameters: the height in the longitudinal direction and the depth in the radial direction, as shown in Figure 4. The depth can be determined through the effective radius method. The height can be determined by the temperature data acquired by the fiber optic sensor. Assuming that whenever the fiber optic sensor passes the boundary of a defect, the slope of the temperature distribution plot will be the largest. After calculating the slope of the plot in the region that has a temperature anomaly caused by necking, there is a point that intercepts with the x-axis that is the center of the temperature anomaly. As shown in Figure 6, there are two points with the lowest and highest changing rate. The locations of these two points determine the upper and lower boundary of the necking. The gap between the boundaries is the portion of the optical fiber passing through the necking region. By knowing the location and the size of the temperature drop in Figure 4, all the parameters are available to reconstruct the defect. Usually, the larger the height of the necking is, the larger the opening of the temperature drop is; the larger the depth of the necking is, the lower the maximum temperature drop is (Figure 4). The inverse modeling shows the lower boundary of necking is 7.73 m and the upper boundary is 7.29 m distant from the shaft top. The depth of necking (Figure 4) is 189 mm. After reconstruction of the defect, the temperature distribution is extracted. The comparison between the temperature distribution of the forward modeling and inverse modeling is presented in Figure 7. The temperature distribution between the forward modeling and inverse modeling agrees well with less than 1% error.

### 3.2. Void Defect

#### 3.2.1. Forward Modeling

**Figure 8:**
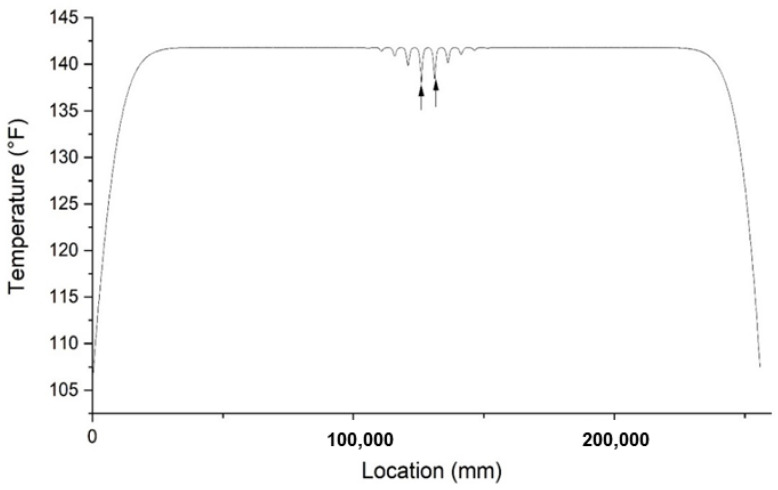
Temperature distribution for a void defect.

When it has a void defect, a shape of rectangular cuboid is assumed in this study. This shape of void defect is consistent with the tied sand bags as used in TIP field studies [1,7]. The center of the defect is at location (0, −787.4 mm, −7500 mm) in the Cartesian coordinate system. The origin point (0, 0, 0) of our defined Cartesian coordinate system is the center of the circular cross section on the top of the shaft; z is in the longitudinal direction of the shaft (Figure 1). The defect is a 254 mm × 254 mm × 400 mm rectangular cuboid-shaped cutoff from the shaft. The temperature distribution of the rectangular-cuboid void of forward modeling is shown in Figure 8. The temperature distribution of a void defect consists of several local minima. The void only affects the temperature distribution of vicinity regions and the temperature distribution on the opposite side of the shaft remains unchanged. Thus, the temperature between local minima in Figure 8 is as normal as the intact part of the shaft. This feature can be used to separate a necking defect from a void defect.

#### 3.2.2. Inverse Modeling

**Figure 9:**
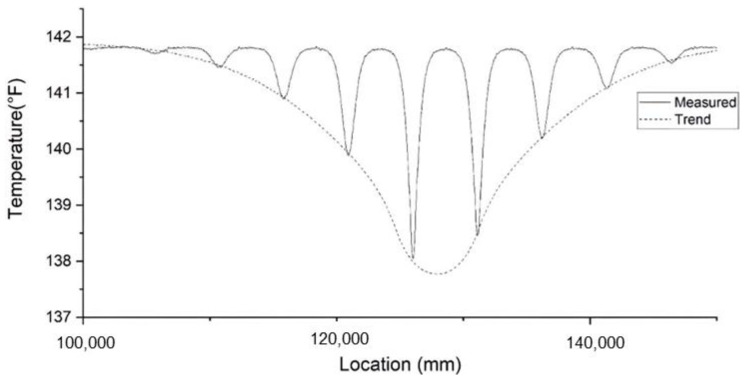
Trend line connecting each bottom point for a void defect.

To determine the location of a void, the method that applies to necking can also be considered. Although the plot of the temperature distribution of the void has more than one local temperature minimum, the location that has the lowest temperature measurement can still be an indicator of the center of the void. Fiber optic sensors may not be able to measure the temperature at the center of the void. Therefore, a trend line is drawn by connecting each local minimum to fit a similar plot to necking. An example of a fitted trend line is shown in Figure 9. The point that has the lowest temperature determines the z coordinate of the defect location. The *x* and *y* coordinates are determined by the location of the lowest minima.

**Figure 10:**
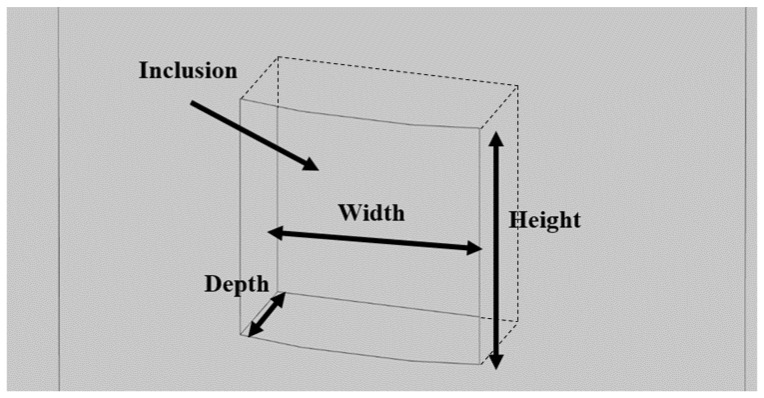
Sketch of void defect for forward and inverse modeling.

**Figure 11:**
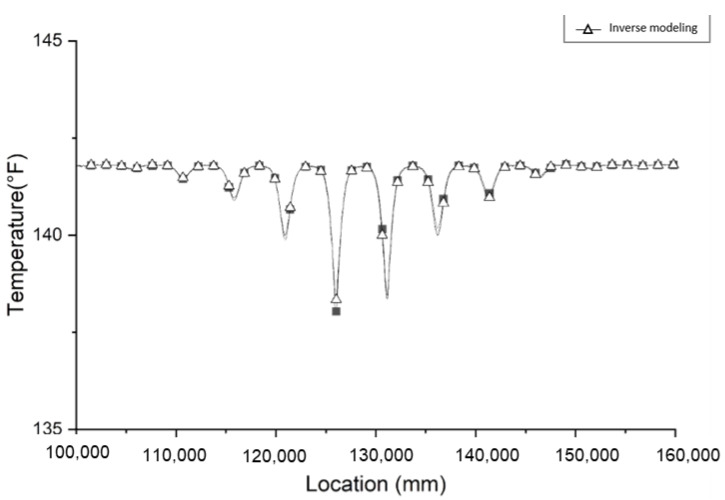
Comparison of temperature distribution between forward and inverse modeling of void defect.

Besides the depth and height, the width at the tangent direction of the shaft is also needed to determine the size of a void, as shown in Figure 10. The effective radius method is less accurate when estimating the size of a void defect. The result usually underestimates the effective radius of the defective cross section of a shaft. Thus, a new method may be needed when estimating the size of a void. The height of a void can be calculated with a similar method to the one for necking, as shown in Figure 6. By applying the method for necking on the fitted trend line as shown in Figure 9, the upper and lower boundaries of the void can be obtained. By applying the temperature changing rate analysis to the drawdown curve of the smallest local minimum, we can obtain two points at the lowest and the highest changing rate. The distance between these two points determines the width of the void with the center of the void determined as above. As for the depth, it is assumed to be equal to the width to simplify the calculation. Then, the estimated defect is input into the model and a simulation is run. Based on the result, we keep the width unchanged, and modify the depth according to the result. If the simulation result shows lower minima, the width size is reduced and the simulation runs again, and vice versa. The procedures repeat until the simulation result is close to the real data. Our final inverse model has a defect of which the size is 270 mm × 169 mm × 450 mm with the center location at (−5 mm, −829.9 mm, −7503 mm). The comparison at the damaged region between forward and inverse modeling is shown in Figure 11. It should be noted that the inverse modeling we develop in this paper is to reconstruct the location and dimensions of the defect of forward modeling approximately. Due to the essential mechanism of inverse modeling, it is unlikely to reconstruct the defect exactly as prescribed in the forward modeling.

**Figure 12:**
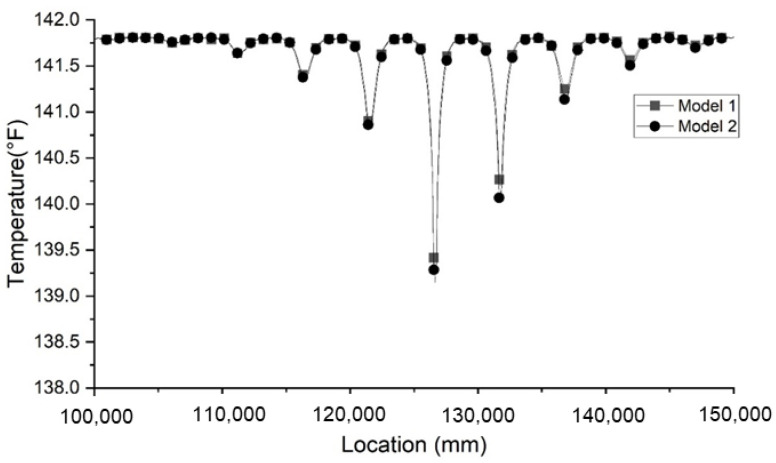
Comparison of two models with different dimensions of defects but the same volume.

At the defective cross section of the shaft, this method only determines the effective cross-section area rather than the actual geometry of the void. Two models of rectangular cuboid void are established with different length–width ratios and similar effective cross-section areas. The other defect prescribed is a 396 mm × 115 mm × 450 mm cutoff with the center location at (0, −856.9 mm, −7500 mm). As shown in Figure 12, two models have a similar temperature distribution within the shaft. It demonstrates that void defects that have the same cross-section area can cause similar temperature anomalies. The estimated geometry may not be the exact same as its original geometry for most of inverse problems that do not have unique solutions. Tangent size determined using the above-mentioned method is most accurate when fiber optic sensors pass the exact center of the defect. However, in most situations, optical fibers cannot directly measure the temperature distribution at the center of the defect. In these situations, the tangent size determined by the plot is usually overestimated. The overestimated tangent size of the defect may further affect the estimation of the radial size of the defect. Overall, this method provides an acceptable prediction of area loss on the horizontal plane and size in the vertical direction. Further study is needed to improve this method of interpretation. It should be noted that this interpretation method may not represent the exact geometry of the void defect by only assuming a rectangular cuboid shape of the void. The results may be different when different shapes of void defect are encountered.

## 4. Conclusions

Fiber optic sensing can greatly enhance the spatial resolution of temperature measurement in engineering applications. However, its application in TIP cannot excel without innovation in the fiber deployment and corresponding inverse modeling for defected shaft reconstruction. The proposed spiral fiber deployment and corresponding inverse modeling in this paper not only improve the defect location prediction but also give the shape prediction, especially for void defects. Based on this study, we can conclude:

By our proposed fiber optic-based TIP, the optical fiber is tied spirally around the reinforcement cage. A cylindrical surface temperature distribution inside the drilled shaft can be obtained. Therefore, the location and shape of defects can be predicted beyond the effective radius method used in the conventional TIP for a drilled shaft.

For necking defects, the inverse modeling for the size prediction in the radial direction shows good agreement with the forward modeling results, and therefore a relatively good prediction of the defect.

For void defects, one more parameter is needed to determine the size of the defect, which is the size at the tangent direction of the shaft. By connecting the local minima, it can result in a trend line similar to the plot of a necking defect. The defect prediction based on this trend line for inverse modeling shows good agreement with the initial setup of forward modeling.

Different voids with similar cross-section areas could result in similar temperature distributions. In most cases, overestimation of the tangent direction and underestimation of the radial direction may happen. To obtain a better void prediction, the current proposed method to determine the geometry of the defect needs to be improved.

This work can contribute to the further evolution of the TIP from the nondestructive testing stage to the quantitative nondestructive evaluation stage by better helping the prediction of the integrity of cast-in-place drilled shafts. Based on this better prediction of the location and shape of defects in drilled shafts, the decision maker can be more informed. The technology of fiber optic-based TIP is still at the development stage, and the method of inverse modeling is rather explorative. The field testing of this proposed technology is needed in the future to further improve this method of inverse modeling and installation method of optical fibers. Due to the tough environment during the shaft construction, it is suggested that the optical fiber be put in metal protection sleeves with multiple threads to prevent breakage.

## Figures and Tables

**Table 1:** Soil and Concrete Properties [29,30].

**Soil Properties**	**Unit**	**Value**
Density	kg/m^3^	1800
Soil solid thermal conductivity	W/(m·K)	5
Water thermal conductivity	W/(m·K)	0.5
Air thermal conductivity	W/(m·K)	0.05
Soil solid heat capacity	J/(kg·K)	850
Water heat capacity	J/(kg·K)	4190
Porosity	%	51.1
Water Content	%	39.8
Saturation	%	97
**Concrete Properties**	**Unit**	**Value**
Density	kg/m^3^	2300
Thermal conductivity	W/(m·K)	1.8
Heat capacity	J/(kg·K)	880
Heat generation rate	$W/m^{3}$	2137.2e^−0.9t^ (t is in days)

## Data Availability

Data is contained within the article.

## References

[1] Mullins G. (2010). Thermal integrity profiling of drilled shafts. DFI J. J. Deep Found. Inst..

[2] Johnson K.R. (2016). Analyzing thermal integrity profiling data for drilled shaft evaluation. DFI J. J. Deep Found. Inst..

[3] Mullins G., Winters D. (2011). Infrared Thermal Integrity Testing Quality Assurance Test Method to Detect Drilled Shaft Defects.

[4] Mullins G. (2013). Advancements in drilled shaft construction, design, and quality assurance: The value of research. Int. J. Pavement Res. Technol..

[5] Boeckmann A.Z., Loehr J.E. (2019). Evaluation of thermal integrity profiling and crosshole sonic logging for drilled shafts with concrete defects. Transp. Res. Rec..

[6] Winters D. In Comparative Study of Thermal Integrity Profiling with Other Nondestructive Integrity Test Methods for Drilled Shafts. Proceedings of the Geo-Congress 2014: Geo-Characterization and Modeling for Sustainability.

[7] Schoen D.L., Canivan G.J., Camp W.M. Evaluation of Thermal Integrity Profiling (TIP) Methods–Probe, Embedded Wire and Wire Suspended in CSL Tubes. Proceedings of the IFCEE 2018.

[8] Rui Y., Kechavarzi C., O’Leary F., Barker C., Nicholson D., Soga K. (2017). Integrity testing of pile cover using distributed fibre optic sensing. Sensors.

[9] Soga K., Luo L. (2018). Distributed fiber optic sensors for civil engineering infrastructure sensing. J. Struct. Integr. Maint..

[10] Minardo A., Bernini R., Amato L., Zeni L. (2011). Bridge monitoring using Brillouin fiber-optic sensors. IEEE Sens. J..

[11] Regier R., Hoult N.A. (2014). Distributed strain behavior of a reinforced concrete bridge: Case study. J. Bridge Eng..

[12] Webb G., Vardanega P., Hoult N., Fidler P., Bennett P., Middleton C. (2017). Analysis of fiber-optic strain-monitoring data from a prestressed concrete bridge. J. Bridge Eng..

[13] Xu J., Dong Y., Zhang Z., Li S., He S., Li H. (2016). Full scale strain monitoring of a suspension bridge using high performance distributed fiber optic sensors. Meas. Sci. Technol..

[14] Damiano E., Avolio B., Minardo A., Olivares L., Picarelli L., Zeni L. (2017). A laboratory study on the use of optical fibers for early detection of pre-failure slope movements in shallow granular soil deposits. Geotech. Test. J..

[15] Hong C.-Y., Yin J.-H., Zhang Y.-F. (2016). Deformation monitoring of long GFRP bar soil nails using distributed optical fiber sensing technology. Smart Mater. Struct..

[16] Sun Y., Shi B., Zhang D., Tong H., Wei G., Xu H. (2016). Internal deformation monitoring of slope based on BOTDR. J. Sens..

[17] Xu D.-S., Yin J.-H. (2016). Analysis of excavation induced stress distributions of GFRP anchors in a soil slope using distributed fiber optic sensors. Eng. Geol..

[18] Schwamb T., Elshafie M.Z., Soga K., Mair R.J. (2016). Considerations for monitoring of deep circular excavations. Proc. Inst. Civ. Eng. Geotech. Eng..

[19] Schwamb T., Soga K. (2015). Numerical modelling of a deep circular excavation at Abbey Mills in London. Géotechnique.

[20] Cheung L., Soga K., Bennett P.J., Kobayashi Y., Amatya B., Wright P. (2010). Optical fibre strain measurement for tunnel lining monitoring. Proc. Inst. Civ. Eng. Geotech. Eng..

[21] Alhaddad M., Wilcock M., Gue C., Bevan H., Stent S., Elshafie M.Z., Soga K., Devriendt M., Wright P., Waterfall P. Multi-suite monitoring of an existing cast iron tunnel subjected to tunnelling-induced ground movements. Proceedings of the Tunneling and Underground Construction.

[22] Luo M., Liu J., Tang C., Wang X., Lan T., Kan B. (2019). 0.5 mm spatial resolution distributed fiber temperature and strain sensor with position-deviation compensation based on OFDR. Opt. Express.

[23] Zhong R., Guo R., Deng W. (2018). Optical-fiber-based smart concrete thermal integrity profiling: An example of concrete shaft. Adv. Mater. Sci. Eng..

[24] Schindler A.K., Folliard K.J. (2005). Heat of hydration models for cementitious materials. ACI Mater. J..

[25] Liu W., He P., Zhang Z. (2002). A calculation method of thermal conductivity of soils. J. Glaciol. Geocryol..

[26] Sáez Blázquez C., Farfán Martín A., Martín Nieto I., Gonzalez-Aguilera D. (2017). Measuring of thermal conductivities of soils and rocks to be used in the calculation of a geothermal installation. Energies.

[27] Abu-Hamdeh N.H. (2003). Thermal properties of soils as affected by density and water content. Biosyst. Eng..

[28] Winters D., Mullins G. In Thermal Integrity Profiling of Concrete Deep Foundations. Proceedings of the Geo-Construction Conference/ADSC Expo 2012.

[29] Young R., Warkentin B. (1975). Soil Properties and Behaviour.

[30] Neville A.M. (2011). Properties of Concrete.

[31] Iskander M., Roy D., Kelley S., Ealy C. (2003). Drilled shaft defects: Detection, and effects on capacity in varved clay. J. Geotech. Geoenviron. Eng..

